# Pulseless electrical activity and successful out-of-hospital resuscitation – long-term survival and quality of life: an observational cohort study

**DOI:** 10.1186/1757-7241-20-74

**Published:** 2012-10-30

**Authors:** Sini Saarinen, Antti Kämäräinen, Tom Silfvast, Arvi Yli-Hankala, Ilkka Virkkunen

**Affiliations:** 1 Department of Anaesthesia and Intensive Care, Helsinki University Central Hospital, Helsinki, Finland; 2 Emergency Medical Service, Tampere University Hospital, Tampere, Finland; 3 Department of Anaesthesiology, Medical School, University of Tampere, Tampere, Finland; 4 Department of Surgery and Anaesthesia, Tampere University Hospital, Tampere, Finland

## Abstract

**Background:**

The aim of the study was to evaluate the long-term outcome of patients successfully resuscitated from pre-hospital cardiac arrest with initial pulseless electrical activity (PEA), because the long-term outcome of these patients is unknown. Survival, neurological status one year after cardiac arrest and self-perceived quality of life after five years were assessed.

**Methods:**

This retrospective study included adult patients resuscitated from PEA between August 2001 and March 2003 in three urban areas in southern Finland. A validated questionnaire was sent to patients while neurological status according to the Cerebral Performance Category (CPC) -classification was assessed based on medical database notes recorded during follow-up evaluations.

**Results:**

Out of 99 included patients in whom resuscitation was attempted, 41 (41%) were successfully resuscitated and admitted to hospital. Ten (10%) patients were discharged from hospital. Seven were alive after one year and six after five years following cardiac arrest. Five of the seven patients alive one year after resuscitation presented with the same functional level as prior to cardiac arrest.

**Conclusions:**

Patients with initial PEA have been considered to have poor prognosis, but in our material, half of those who survived to hospital discharge were still alive after 5 years. Their self-assessed quality of life seems to be good with only mild to moderate impairments in activities of daily life.

## Background

In Europe, the estimated incidence of out-of-hospital cardiac arrest (OHCA) with any initial rhythm is 35–37/100 000/year [1,2]. Although ventricular fibrillation (VF) is still the most common initial rhythm in OHCA, its incidence has been constantly declining. Previous studies performed in the 1980s have reported the initial rhythm to be VF in 61–65% in OHCA, while during the last ten years VF has been initial rhythm in 35–48% of OHCA [1-4]. Concomitantly, the proportion of pulseless electrical activity (PEA) has increased, currently ranging between 22 to 30% in OHCA [4-7]. This indicates PEA to occur 8–11/100 000/year in Europe [1,4-7]. In Finland, studies have reported the incidence of PEA to vary from 4 to 20/100 000/year [4,7].

PEA is associated with a better prognosis than asystole but worse than that of VF. The survival rates to hospital discharge are approximately 4–7%, 2% and 17–21%, respectively [1,7-10]. Incidences for hospital discharge are estimated to be 3.6/100 000/year for VF patients and 2.2/100 000/year for all-rhythm OHCA [1]. The prognosis of patients with initial PEA is better if the delay to return of spontaneous circulation (ROSC) is short [7]. Better prognosis compared to asystole could be partly explained by the fact that some patients with PEA have undetectable but minimally perfusing circulation preserved which can only be detected by invasive monitoring or with ultrasound imaging. Patients with pseudo-PEA carry a better prognosis than patients presenting with electrical activity without myocardial contractions [8].

Recent studies have shown that 5.8–6.8% of PEA patients are alive 30 days after resuscitation [7,11]. The long-term outcome of patients resuscitated from PEA is unknown, whereas the long-term outcome of patients with VF as initial rhythm is well documented [12]. Because the percentage of PEA as initial rhythm in cardiac arrest (CA) is increasing, the short-term outcome is better than previously documented and there is a lack of long-term outcome data, we aimed to investigate the survival of PEA patients 1 and 5 years after cardiac arrest. We also wanted to assess their self-perceived quality of life at present, more than five years after resuscitation, and to determine whether their Cerebral Performance Category (CPC) - class [13] had changed one year after resuscitation.

## Methods

The present study included patients from three emergency medical service (EMS) systems in southern Finland, the paramedic-staffed EMS system in the city of Tampere and the physician-staffed helicopter EMS (HEMS) systems in Helsinki and Turku areas, between August 2001 and March 2003. For clarity, the Helsinki area HEMS serves areas surrounding the city of Helsinki, and the city itself is covered by a separate EMS system not included in this study. In Finland, majority of physicians in HEMS systems are specialists in anaesthesiology and intensive care, whereas paramedics undergo 3–4 years of education in emergency medicine.

Originally, the data were collected prospectively for a study with a focus on regurgitation during resuscitation regardless of the initial rhythm [6]. The same database was now retrospectively used for this study, which is a post hoc-analysis with a focus on the long-term outcome of patients with PEA.

All consecutive patients more than 16 years of age, who suffered an OHCA of presumed cardiac origin with PEA as the initial cardiac rhythm and in whom resuscitation was attempted were included. PEA was defined as monitored electrical activity with no detectable pulse. As defined in the 2004 Utstein guidelines [14], the cause of arrest was presumed to be of cardiac origin when no external cause such as trauma, intoxication, airway obstruction, drowning or haemorrhage was evident. Patients with a disease at a terminal stage, e.g. end-stage malignancy, were excluded. Dispatch centre personnel provided basic life saving instructions to caller if CA was recognized. Patients with PEA were treated according to current guidelines during the study period [15]: endotracheal intubation was used to secure airway and epinephrine was given in 1mg boluses every 3–5 minutes and possible subsequent VF was defibrillated. As a specific treatment for suspected pulmonary embolism causing PEA, all EMS systems were able to provide thrombolysis. At the time of the study period, therapeutic hypothermia was not routinely provided for these patients.

In the present study, assessment of long-term survival was performed after 1 and 5 years following OHCA. One of the authors (SS) evaluated the premorbid CPC and CPC one year after OHCA retrospectively based on patient medical records. The CPC –classification is a five-stage scale of neurological state [13]. Class 1 corresponds to good cerebral performance with no or only mild neurologic or psychological defect, class 2 corresponds to moderate cerebral disability with sufficient cerebral function for independent activities of daily life. Class 3 indicates severe cerebral disability with dependence on others for daily support because of impaired cerebral function. Class 4 stands for coma or vegetative state without interaction with the environment and Class 5 means brain death. Briefly, classes 1–2 correspond to sufficient cerebral function for independent activities of daily life, while classes 3–5 reflect dependency on others. We estimated whether a long-term change in neurological status using the CPC-classification had occurred after OHCA with PEA as the initial rhythm.

The National registry of Statistics Finland was used to evaluate the time and cause of death (COD) in the non-surviving victims of pre-hospital PEA. The patients' medical records from receiving hospitals were used to obtain the cause of OHCA of patients who survived until follow-up.

We sent a fifteen dimensional (15D) questionnaire of health-associated quality of life to the long-term survivors [16]. The 15D-questionnaire is validated in the Finnish National Centre for Health Program Evaluation and includes 15 questions describing self-assessed performance in activities of daily life [16,17]. If a patient was unable to answer the questionnaire because of disability, his/her next of kin was asked to fill the form in co-operation with the patient.

The study protocol was approved by the ethical review board of the Helsinki University Hospital.

Statistical analyses were performed using the SPSS for Windows V16.0-software (SPSS Inc., Chicago, IL, USA). Chi-Square Test was used for categorical variables. Statistical significance was set at p <0.05. The data are presented as mean ± SD unless otherwise indicated. 95% confidence intervals (CI) were calculated for proportions.

## Results

### Study population

During the twenty-month study period, resuscitation was attempted in 452 OHCA patients, out of which 117 (26%, CI 22–30%) had PEA as initial rhythm [6]. During data analysis, eighteen (15%, CI 10-23%) patients with either an external cause of PEA [trauma (7), intoxication (5)] or end-stage malignancy (6) were detected. These patients with an obvious external cause of arrest were supposed to be excluded originally with the intent to evaluate arrests of cardiac origin and were thus excluded from further analysis. In cases when CPR was initiated despite the presence of end-stage malignancy, this was due to the lack of information regarding the malignant disease and therefore patients were excluded retrospectively due to apparent futility. Thus, the study population eventually consisted of 99 patients. Their mean age was 69 years, and 30 (30%, CI 22–40%) were women. The most common aetiologies behind PEA were cardiac disease in 49 (49%, CI 40–59%), aortic disease in 12 (12%, CI 7–20%) and pulmonary embolism in 9 (9%, CI 5–17%) patients.

### Survival

**Figure 1:**
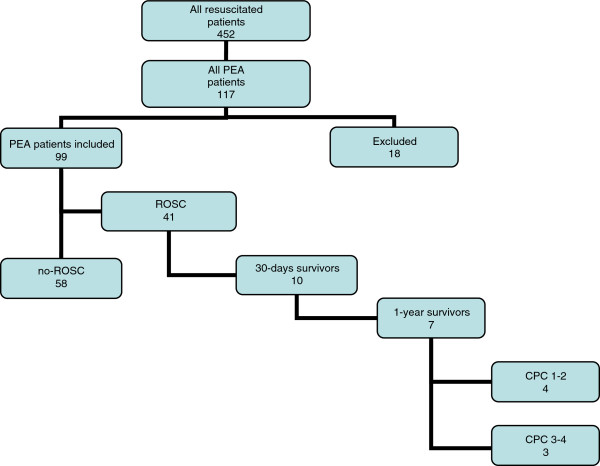
**Survival and neurological recovery of PEA patients.** PEA patients survival to ROSC, 30-days and 1-year survival and neurological state described with CPC classification one year after resuscitation. CPC 1–2 correspond to sufficient cerebral function for independent activities of daily life, while classes 3–5 reflect dependency on others. PEA= pulseless electrical activity, ROSC= return of spontaneous circulation, CPC= cerebral performance category.

Forty-one (41%, CI 32–51%) patients regained spontaneous circulation (ROSC). None of the admitted patients were treated with therapeutic hypothermia. Ten (10%, CI 5-18%) patients were discharged from hospital and alive after 30 days, and seven (7%, CI 3–14%) were still alive one year after resuscitation (Figure 1). Regarding the delay to ROSC, no difference was observed between non-survivors, patients who survived less than 30 days and long-term survivors. The delays to ROSC in association with short and long-term survival are presented in Table 1. In patients who regained ROSC, most frequent aetiologies were cardiac disease in 20 (49%, CI 34–64%), unknown in 6 (15%, CI 7–29%) and neurological disease in 5 patients (12%, CI 5–26%).

**Table 1:** ROSC and survival

**Survival status**	**Alive**	**Alive**	**Alive**	**p**
	**<30 days (n= 31)**	**>30 days (n=10)**	**>1 year (n=7)**	
Mean delay to ROSC (±SD)	23 (±15) min	20 (±8) min	20min (±8) min	NS

Mean delays to ROSC (±SD) with PEA patients who survived less than 30 days, over 30 days or over a year after CA. Comparison of delay to ROSC and survival between all groups. ROSC= return of spontaneous circulation, PEA= pulseless electrical activity, CA= cardiac arrest, NS= not significant.

### Quality of life

One year after resuscitation, the neurological status of four of the seven survivors corresponded to CPC classes 1–2 (sufficient cerebral function for independent activities of daily life) whereas three of them were categorised to CPC 3–4. In five of the seven one-year survivors the CPC-class was the same as before resuscitation.

Six patients (6% CI 3–13%) were still alive 5 years after cardiac arrest, and five of them were still alive in spring 2009. They responded to the 15D-questionnaire of health-associated quality of life 6.5 to 7.5 years after CA. Next of kin of two patients answered to the questionnaire on behalf of the patient. Patients assessed 65 of 75 (87%, CI 77–93%) estimated properties as normal or mildly impaired. All patients reported normal or only mildly impaired function in seeing, hearing, sleeping, eating, speech and in urination or defecation. Levels of energy, distress and depression were also estimated to remain normal or mildly worsened. Patients with a CPC status of 2 or 3 before CA reported moderate impairment in functional level, need for help or total lack of capability to perform independently occupationally or in leisure activities, psychical functions, mobility and in sexual life.

## Discussion

In this study, we observed that almost half (41%, CI 32–51%) of the OHCA patients who presented with PEA due to a presumed cardiac cause in the pre-hospital setting survived to hospital admission, and 10% (CI 5–18%) of them were discharged alive and alive after 30 days. These figures are relatively high compared to other studies. In the Helsinki city EMS system, 25.7% of out-of-hospital PEA patients survived to hospital admission and 5.8% were alive after 30 days [7]. A recent systematic review of OHCA in Europe reported a hospital discharge rate of 9% for all initial rhythms and 19% for VF [2]. Half of our patients who survived to hospital discharge were still alive after 5 years.

Our material included PEA patients in whom resuscitation was attempted and who did not have external or apparently futile cause for PEA, which could be possible explanations for high survival rates. In regard to the focus of the initial study [6], this material represents a consecutive subgroup of OHCA patients treated by three distinct systems, rather than a population based evaluation of PEA incidence and survival. Therefore the study material was not collected according to the criteria set in the Utstein guidelines [14] and the accurate incidence of OHCA or PEA as the initial rhythm, or survival per 100 000 per year cannot be accurately presented. The reported rates for overall OHCA incidence and survival in Tampere and Helsinki city have been reported to be 46–67/100 000/yr and 13–19.6%, respectively [18,19]. To our knowledge, a specific report conforming to the Utstein style on the incidence and survival rates in the Turku area has not been published.

Recent review of quality of life after CA shows that usually patients report their quality of life to be good [20]. According to the 15D- questionnaire, long-term survivors in our study also seem to have recovered quite well, have a good quality of life and some of them are even able to work. Harve et al. investigated the quality of life after CA with VF/VT (ventricular tachycardia) [12]. All 10 patients in their study were independent in their activities of daily life, 4 had mild cognitive problems. However, most patients' self-perceived quality of life after CA seems to be good despite of the initial rhythm. Lack of standardised tool to evaluate CA patients' quality of life makes comparison between studies difficult, since multiple different questionnaires and research methods are being used at the moment. Creating a standardised research method specially designed to evaluate quality of life after CA becomes crucial in future [20].

The above findings suggest that the prognosis of patients who present with PEA in the out-of-hospital setting is not as dismal as generally considered. Putting these results in perspective with the epidemiological figures of OHCAs in Europe is interesting. If the annual rate of all-rhythm CAs in Europe is 38/100 000 persons and the proportion of PEA is close to 30%, with a European population of 731 000 000 this would annually equal to approximately 83 000 victims of PEA [1]. With one and five year survival rates of 7% and 6%, the figures on a European scale would equal to 5 000–5 800 long-term survivors. The observed outcome rates were achieved without the use of therapeutic hypothermia. The current European Resuscitation Council Guidelines for Resuscitation recommend the use of therapeutic hypothermia for comatose survivors of cardiac arrest regardless of the initial rhythm in CA, when active post resuscitation care is considered appropriate [21].

In our material, cardiac aetiology was not associated with better rates of ROSC. Patients in whom ROSC was gained had more often neurologic aetiology (p=0.031) and less often aortic aetiology (p=0.026). In 5-year survivors the underlying cause of PEA was cardiac in three patients and other aetiologies were pulmonary embolism, pulmonary and unknown. The association of aetiology with short- and long-term prognosis is another area of interest considering the wide variety of underlying aetiologies – as well as limited intensive care and hospital resources. This could be a potential aim of larger prospective studies in future, as our material is too small to reveal reliable associations between outcome and aetiology.

There are some limitations related to this study. The study setting was partly retrospective in that the patients were retrospectively identified. Some patients may have been excluded from the study as EMS personnel may have forgotten to fill the documentation form after CPR or the documentation may have been lost during the tracking process. The number of interviewed survivors was small, but the focus of that part of the study was not to compare but rather to assess the quality of life. CPC classification is commonly used to evaluate neurological survival after CA [20], but it is a rough scale and neuropsychological tests would give more detailed information. By definition, the 15D- questionnaire is a validated tool for assessment of the quality of life regardless of the underlying health state. As a subjective report, it is feasible also among cardiac arrest survivors. We did not include all patients with PEA as initial cardiac rhythm. The main reason to include only patients with presumed cardiac aetiology was to exclude those with immediately observed futility, such as traumatic cardiorespiratory arrest or malignant disease underlying OHCA. However, keeping these limitations in regard, these results suggest that the resuscitation of patients with PEA as the initial rhythm can yield good long term results even with over 20 min delay to ROSC.

## Conclusions

Half of the patients surviving to hospital discharge were alive 5 years after OHCA and reported a good self-perceived state of recovery. The documentation of moderately favourable chances for good long-term outcome of PEA patients might encourage receiving hospitals to use more intensive treatment options, such as utilisation of therapeutic hypothermia.

## Competing interests

The authors declare that they have no competing interest.

## Authors' contributions

SS processed and analysed the data and drafted the manuscript. AK involved in conception of the study design, data analysis and revising the manuscript. TS and AY gave assistance to analysing the data and revised the manuscript. IV collected the data, involved in conception of the study design and revised the manuscript. All authors have given the final approval of this article to be considered for publication. All authors read and approved the final manuscript.
